# Pediatric Graves’ Disease: Challenges Associated With Thyroidectomy and Perioperative Management in a Three-Year-Old Child

**DOI:** 10.7759/cureus.79893

**Published:** 2025-03-01

**Authors:** Emad Ramadan, Saad Nadeem, Muhammad Zain, Sharif Mohamed

**Affiliations:** 1 Anesthesiology, University of Texas Medical Branch, Galveston, USA; 2 Medicine, Texas A&M College of Medicine, Houston, USA; 3 Internal Medicine, University of Houston, Houston, USA

**Keywords:** airway management, endocrine stabilization, exophthalmos, graves' disease, neck hematoma, pediatric hyperthyroidism, perioperative care, thyroidectomy

## Abstract

Graves’ disease is a rare cause of hyperthyroidism in children, and it poses specific problems in diagnosis and surgery. We report a three-year-old female child who had Graves’ disease with exophthalmos, for which she underwent a total thyroidectomy. On the preoperative assessment, the patient had tachycardia, hypertension, and severe bilateral proptosis. Managing the airway was challenging due to a shift in the trachea and possible tracheomalacia. Endocrine stabilization, protection for the eyes during surgery, and close observation for thyroid storms and neck hematoma were some strategies employed during the perioperative period. This case highlights the importance of tailored, multidisciplinary approaches to optimize outcomes in pediatric thyroidectomy.

## Introduction

Graves’ disease, one of the most common causes of hyperthyroidism, is an autoimmune disorder characterized by the overproduction of thyroid hormones [1]. The condition typically presents between the ages of 20 and 30, though it is extremely rare in young children. The estimated incidence is only one case per 1,000,000 children under four years [2]. Diagnosing Graves' disease in this age group can be challenging due to the typical symptoms, which include exophthalmos, goiter, heat intolerance, and hyperactivity. These signs are often mistaken for other pediatric conditions. The pathogenesis involves the production of thyroid-stimulating immunoglobulins, which mimic the action of the thyroid-stimulating hormone (TSH) and lead to excessive thyroid hormone production. Imaging studies and thyroid receptor antibody testing usually confirm the diagnosis, with results typically showing suppressed TSH levels and elevated free T4 and T3 [3].

Treatment options for Graves' disease include radioactive iodine therapy, antithyroid medications, and surgery. Each approach must be carefully considered to balance the effective management of hyperthyroidism with the potential risks to a child’s development, as thyroid hormones play a critical role in growth. If left untreated, Graves’ disease can severely impair a child’s growth and development [2,3].

## Case presentation

Our patient was a three-year-old female child with a diagnosis of hyperthyroidism secondary to Graves’ disease and exophthalmos. She had a past medical history of iron deficiency with pica, controlled with ferrous sulfate, and a previous COVID-19 infection in October 2020, from which she recovered uneventfully at home. The patient was scheduled for a total thyroidectomy under general anesthesia on April 23, 2021. Preoperative vital signs showed tachycardia (heart rate, HR, 112 beats per minute) and hypertension (blood pressure 138/78, >99th percentile). The patient also had a dry cough and clear rhinorrhea, attributed to allergies by the mother. Laboratory results revealed elevated white blood cell and platelet counts. Physical examination findings included moderate bilateral proptosis, enlarged tonsils, and no palpable neck mass. The airway exam did not document a Mallampati score, but no external airway abnormalities were noted. A preoperative chest X-ray was performed on April 15, 2021, and the impression was normal, with no evidence of lower respiratory tract involvement. The patient’s lab values were consistent with hyperthyroidism (Table 1).

**Table 1:** Thyroid lab values TSH: thyroid-stimulating hormone; IU: international units

Test	Value	Reference range
TSH	0.02 mIU/L	0.4-4 mIU/L
Free T3	18.8 pg/mL	2-4.4 pg/mL
T4 total	>24.9 mcg/dL	5-12 mcg/dL
T4 free	>6.99 ng/dL	0.8-2 ng/dL

While atenolol and Lugol's solution were listed as part of the patient’s outpatient medications, the anesthesia record does not specify whether they were administered in the immediate preoperative period. Anesthesia was induced via inhalational induction with sevoflurane. After induction, a peripheral IV was placed, and adjuncts, including lidocaine 1% (2 mL), were administered. Two intubation attempts were made. The first attempt, performed by the resident (A.F.) under the supervision of the attending anesthesiologist (S.M.), involved the use of lidocaine spray to reduce airway reactivity, but the attempt was unsuccessful due to poor visualization of the glottis. The second attempt, performed by the same team, successfully used video-assisted laryngoscopy with a Macintosh #2 blade. A 5.0-mm oral cuffed endotracheal tube (ETT) with a nerve integrity monitor (NIMS) was placed and secured at 16 cm from the incisors. The NIMS tube was part of the initial plan to facilitate intraoperative nerve monitoring during the thyroidectomy.

Total intravenous anesthesia was maintained using propofol (85 mg total) and remifentanil (350.88 mcg total) for continuous neuromonitoring, including somatosensory evoked potentials and electromyography of the recurrent laryngeal nerve. Eye protection was prioritized due to the patient’s exophthalmos, with lubrication and taping of the eyes documented in the anesthesia record. Temperature regulation was achieved using a forced air warmer. Intraoperative medications included dexamethasone (8 mg) for antiemetic effects and airway edema prophylaxis, cefazolin (800 mg) for surgical site infection prophylaxis, and esmolol (30 mg) for HR control. Fluid management consisted of 300 mL of lactated Ringer’s solution administered throughout the procedure.

The procedure lasted three hours and 41 minutes. The patient remained hemodynamically stable throughout, with an HR ranging from 100 to 115 bpm. Esmolol was used intermittently to manage transient episodes of tachycardia. Following the procedure, the patient was extubated while awake in the operating room (OR) under controlled conditions. The anesthesia team remained in the OR for 30 minutes after extubation to monitor for any signs of airway compromise, such as stridor, respiratory distress, or tracheomalacia. No complications were observed, and the patient was transferred to the postanesthesia care unit for recovery.

## Discussion

Goiters are known to complicate direct laryngoscopies and intubations among children undergoing thyroidectomy [4-6]. Therefore, it is vital to anticipate potential difficulties with airway maintenance or intubation preoperatively. One of the risk factors in such cases is tracheomalacia, which occurs when the diameter of the trachea collapses to less than half of its normal diameter during negative pressure breathing mechanics, causing difficulty in breathing [4]. Excessive pressure from a goiter on the trachea over a long period can soften its walls, increasing the risk of airway collapse after induction or during extubation. This underscores the need for well-planned airway management throughout the perioperative period. Tracheal deviation is another potential complication that can make intubation more difficult. In this case, the anesthesia record does not explicitly describe tracheal deviation as a contributing factor during intubation. The first intubation attempt was unsuccessful due to poor visualization of the glottis, but the second attempt, using video-assisted laryngoscopy, was successful without further complications.

Thyroid swelling causes airway distortion and poses a risk of endocrine emergencies, leading to widespread endocrine imbalances and metabolic disturbances. Therefore, preoperative and intraoperative endocrine management is essential for successful outcomes in thyroidectomy. Numerous complications may emerge intraoperatively, including the potentially rare but life-threatening condition known as a thyroid storm. Stress surrounding surgery, anesthetic use, and stimulating the thyroid are just a few factors that could trigger a thyroid storm [5]. Most patients with Graves’ disease who are scheduled for surgery are given some form of iodine, e.g., potassium iodide or a saturated solution of potassium iodide, beforehand. This approach is advantageous as it reduces thyroid gland blood flow, vascularity, and intraoperative blood loss [5].

**Figure 1:**
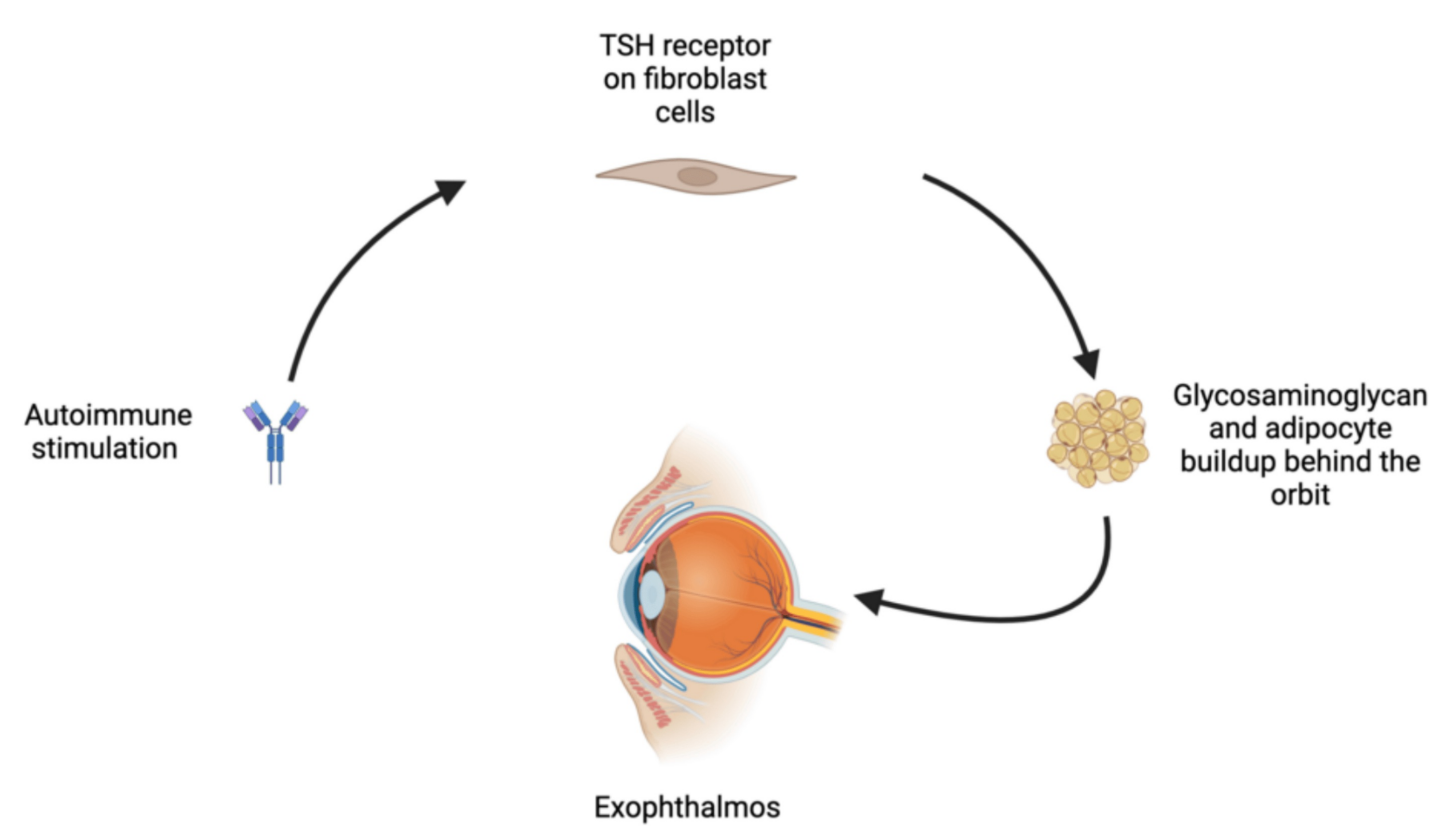
Pathogenesis of exophthalmos TSH: thyroid-stimulating hormone Image credit: This is an original image created by the author Emad Ramadan

Exophthalmos is a condition seen in Graves’ disease patients, resulting in the protrusion of eyeballs. This predisposes the patient to corneal exposure and subsequent abrasions during surgery [6,7]. Adequate eye protection for patients with exophthalmos during the perioperative period is crucial to prevent corneal injuries. While no single method is completely effective, standard intraoperative eye protection measures like eyelid taping, ophthalmic ointment, and eye pads significantly reduce the risk of intraoperative eye injuries [7]. The pathogenesis of exophthalmos in thyrotoxicosis, particularly in Graves' disease, involves the activation of TSH receptors in the orbital fibroblasts. This activation leads to a cascade of events resulting in the buildup of glycosaminoglycans (GAGs) and inflammation. Orbital fibroblasts express TSH receptors, and their activation by TSH or TSH receptor autoantibodies leads to increased production of hyaluronic acid (HA), a type of GAG. The accumulation of HA and other GAGs in the orbital tissues increases osmotic pressure, leading to tissue edema and inflammation (Figure 1). Additionally, the interaction between TSH receptors and insulin-like growth factor-1 receptors (IGF-1R) on orbital fibroblasts further enhances HA production and adipogenesis. This cross-talk between the TSH receptor and IGF-1R amplifies the inflammatory response and tissue remodeling in the orbit [8-10].

Neck hematoma postoperatively is a rare but life-threatening complication after thyroidectomy due to airway compression [11]. Symptoms of a neck hematoma include hoarseness, dysphagia, stridor, and respiratory distress, which can progress to complete airway obstruction if not promptly addressed. A study of 2,320 patients showed an incidence rate of 1.1%, with male patients over the age of 50 being more likely to develop a hematoma postoperatively. Additionally, patients with a history of hypertension faced a higher risk of this complication [11]. Treatment for a neck hematoma requires emergent surgical drainage to relieve airway compression and prevent asphyxiation. In severe cases, immediate airway intervention, such as reintubation or tracheostomy, may be necessary to secure the airway [12].

## Conclusions

This case underscores the clinical significance of personalized perioperative strategies for rare pediatric presentations, emphasizing the role of a tailored, patient-centered approach to improve safety and outcomes in thyroidectomy procedures. The case highlights challenges that arise in the management of Graves’ disease in children undergoing thyroidectomy. Adequate and thorough perioperative evaluation of the patients, appropriate anesthetic plan, and careful intraoperative management are vital for optimizing outcomes. Risks associated with tracheal deviation, hyperthyroidism, and thyroid storm can be mitigated by anticipating airway difficulties, using advanced airway devices, and ensuring endocrine stability. Furthermore, special attention to eye protection strategies in patients with exophthalmos and prompt identification and management of postoperative complications, such as neck hematomas, demonstrates the importance of multidisciplinary care.
